# A Comparative Assessment of Replication Stress Markers in the Context of Telomerase

**DOI:** 10.3390/cancers14092205

**Published:** 2022-04-28

**Authors:** Sabine Meessen, Gregoire Najjar, Anca Azoitei, Sebastian Iben, Christian Bolenz, Cagatay Günes

**Affiliations:** 1 Department of Urology, Ulm University Hospital, 89081 Ulm, Germany; sabine.meessen@medma.uni-heidelberg.de (S.M.); gregoire.najjar@uniklinik-ulm.de (G.N.); anca.azoitei@uniklinik-ulm.de (A.A.); christian.bolenz@uniklinik-ulm.de (C.B.); 2 Department of Dermatology, Ulm University Hospital, 89081 Ulm, Germany; sebastian.iben@uni-ulm.de

**Keywords:** aneuploidy, oncogene-induced replication stress, reactive oxygen species, telomerase, phosho-RPA2, γ-H2AX, 53BP1

## Abstract

**Simple Summary:**

Genetic alterations such as oncogenic- or aneuploidy-inducing mutations can induce replication stress as a tumor protection mechansim. Previous data indicated that telomerase may ameliorate the cellular responses that induce replication stress. However, the mechanisms how this may occur are still unclear. In order to address this question, the accurate evaluation of replication stress in the presence and absence of telomerase is crucial. Therefore, we used telomerase negative normal human fibroblasts, as well as their telomerase positive counterparts to compare the suitability of three protein markers (pRPA2, γ-H2AX and 53BP1), which were previously reported to accumulate in response to harmful conditions leading to replication stress in cells. In summary, we find that pRPA2 is the most consistent and reliable marker for the detection of replication stress. Further, we demonstrated that the inhibition of the DNA-damage activated ATM and ATR kinases by specific small compounds impaired the accumulation of pRPA2 foci in the absence of telomerase. These data suggest that telomerase rescues the cells from replication stress upon supression of DNA damage induction by modulating the ATM and ATR signaling pathways, and may therefore support tumor formation of genetically unstable cells.

**Abstract:**

Aberrant replication stress (RS) is a source of genome instability and has serious implications for cell survival and tumourigenesis. Therefore, the detection of RS and the identification of the underlying molecular mechanisms are crucial for the understanding of tumourigenesis. Currently, three protein markers—p33-phosphorylated replication protein A2 (pRPA2), γ-phosphorylated H2AX (γ-H2AX), and Tumor Protein P53 Binding Protein 1 (53BP1)—are frequently used to detect RS. However, to our knowledge, there is no report that compares their suitability for the detection of different sources of RS. Therefore, in this study, we evaluate the suitability of pRPA2, γ-H2AX, and 53BP1 for the detection of RS caused by different sources of RS. In addition, we examine their suitability as markers of the telomerase-mediated alleviation of RS. For these purposes, we use here telomerase-negative human fibroblasts (BJ) and their telomerase-immortalized counterparts (BJ-hTERT). Replication stress was induced by the ectopic expression of the oncogenic RAS mutant RAS^G12V^ (OI-RS), by the knockdown of ploidy-control genes ORP3 or MAD2 (AI-RS), and by treatment with hydrogen peroxide (ROS-induced RS). The level of RS was determined by immunofluorescence staining for pRPA2, γ-H2AX, and 53BP1. Evaluation of the staining results revealed that pRPA2- and γ-H2AX provide a significant and reliable assessment of OI-RS and AI-RS compared to 53BP1. On the other hand, 53BP1 and pRPA2 proved to be superior to γ-H2AX for the evaluation of ROS-induced RS. Moreover, the data showed that among the tested markers, pRPA2 is best suited to evaluate the telomerase-mediated suppression of all three types of RS. In summary, the data indicate that the choice of marker is important for the evaluation of RS activated through different conditions.

## 1. Introduction

The replication of DNA needs to be balanced in accuracy, speed, distribution, and consumption of the necessary resources such as replication factors (e.g., helicases) and nucleotides. An accurate DNA replication is crucial for the prevention of replication fork stalling (RFS) and replication stress (RS) [1]. DNA replication forks are frequently stalled and have to be overcome for the faithful replication of the entire genomic material [1,2,3,4,5]. Obstacles to the replication machinery can be of intracellular or extracellular origin [1]. Replication stress can be caused by endogenous and exogenous factors that result in high levels of DNA damage and RFS. Endogenous stressors can result from mutations or amplifications that lead to an excessive activity of oncoproteins (oncogene-induced replication-stress: OI-RS) or to the induction of aneuploidy (aneuploidy-induced replication-stress: AI-RS). Similarly, mutations that result in an accumulation of reactive oxygen species (ROS) harm the DNA and cause replication stress (ROS-induced RS). ROS can also accumulate in response to excess metabolic activities or exogenous stressors such as UV light or chemical substances.

Even though replication stress is known as a cause of genomic instability and reduced cell viability, there is no consistent marker that can be used to evaluate the level of replication stress in cells. Although the direct detection of replication dynamics and thus the stalling of replication forks (i.e., replication stress) can be best measured by DNA synthesis using DNA fiber or DNA combing assays [6,7,8,9], these methods are not easy to establish and are time-consuming. Currently, three protein markers are frequently used for the evaluation of replication stress: the replication protein subunit 2 (RPA2), the H2A histone family member X (H2AX), and the Tumor Protein P53 Binding Protein 1 (53BP1) [10,11,12,13,14,15,16].

The replication protein RPA localizes at the sites of replication prior to the initiation of replication and covers the ssDNA track, which is generated by the DNA unwinding helicase Cdc45-Mcm2-7-GINS (CMG) during replication or DNA damage repair. In the case of replication fork stalling, RPA accumulates at the ssDNA and activates the ATR pathway as a stress response [17,18,19,20]. The activation of the ATR kinase triggers a signaling cascade through the recruitment of ATRIP to the site of the extended ssDNA [17]. Upon recruitment, ATR phosphorylates CLASPIN and several other factors, which is in turn required for CHK1 and CHK2 activation [21,22,23,24]. As a response, the cell cycle is delayed through the ATR/Chk1 pathways in order to enable DNA repair. The accumulation of RPA at the sites of replication fork stalling is activated by the phosphorylation of the p32 subunit of the RPA complex [25]. Therefore, pRPA2 has been considered as a suitable marker for determining the level of replication fork stalling and RS [15]. Stalled replication forks can either be restarted or they can collapse, which would result in DNA double-strand breaks (DSBs) and thereby the activation of the DNA damage response (DDR) [16]. To prevent the DSBs, the replication is halted, but the unwinding proceeds [26]. Upon DSBs, which can be induced by irradiation, sustained fork stalling, or other DNA-damaging agents, H2AX is phosphorylated at serine 139 in order to be converted to γ-H2AX [27,28,29]. Therefore, γ-H2AX is used as a molecular marker for the detection of DSBs.

The primary role of γ-H2AX to initiate the DDR pathway in response to endogenous and exogenous DNA-damage has been well-documented during the last two decades [30,31,32,33]. In response to DNA-damage, the kinases ATM- and DNA-PK phosphorylate H2AX, which then initiates the recruitment and localization of DNA-repair proteins to the site of damage. On the other hand, H2AX can be directly phosphorylated by ATR upon replication stress, suggesting that DSB and RS pathways are closely connected [19,20,27,29]. Moreover, H2AX is required for chromatin remodeling, and H2AX phosphorylation increases in response to changes in the chromatin structure during DNA replication and transcription, with different consequences for replicative stress [34,35,36]. An increase of H2AX foci formation was also demonstrated in late S/G2- and M-phase cells after hydroxyurea- and aphidicolin-induced DNA replication stress [37]. However, the suitability of γ-H2AX as a specific marker in response to different sources of RS still needs to be clarified. It has not yet been proven if all replication stress inducers activate ATR and its downstream substrates strongly enough to be detected [38].

The tumor suppressor p53-binding protein 1 is a key player in double-strand break (DSB) repair mechanisms [39]. Depending on the stage of the cell cycle, 53BP1 promotes different repair pathways: during G1, non-homologous end joining (NHEJ) is promoted, whereas in the S and G2 phases, DNA end resection and the homologous recombination (HR) of DSB are promoted. The decision with regard to which way the DSBs are to be repaired is mediated through the interaction partners of 53BP1 (e.g., H2AK15) [39]. Replication stress activates 53BP1, and it has been shown that 53BP1 mediates ATR-CHK1 signaling to protect replication forks under replication stress conditions [40,41]. The 53BP1 that contain nuclear foci mark DNA lesions such as common fragile sites [42,43], and it has been shown that ROS-induced RS increases 53BP1 foci formation at telomeres [16]. Furthermore, 53BP1 foci form in response to OI-RS or AI-RS [44,45].

Telomerase enzymatic activity is crucial to the maintenance of genome integrity by ensuring the complete synthesis of telomeric DNA, thus preventing the loss of genetic material in highly proliferating cells [46,47]. Besides its primary function to compensate for the loss of telomeric DNA, telomerase also has telomere length-independent fundamental biological functions, including DNA damage response, cell survival, and apoptosis [48,49,50,51,52,53,54,55,56,57,58,59,60,61,62]. More recently, emerging data, including studies from our laboratory, have indicated that telomerase activity can suppress replication stress induced by oncogene activation [14], aneuploidy induction [15], or genotoxic reactive oxygen species [16]. More importantly, telomerase activity suppresses the activation of the DNA damage response and alleviates telomeric replication stress, but this effect is independent of telomere length [63]. In each of these studies, a different marker (pRPA2, γ-H2AX, or 53BP1, respectively) was used to determine the impact of telomerase in DDR activation. 

In this study, pRPA2, γ-H2AX, and 53BP1 were compared for their suitability as a replication stress marker. The accumulation of pRPA2, γ-H2AX, and 53BP1 foci formation was evaluated under different replication stress-inducing conditions: AI-RS, OI-RS, and ROS-induced RS. AI-RS was induced by the knockdown of ORP3 or MAD2, the overexpression of an oncogenic RAS variant (RAS^G12V^) induced OI-RS, and for ROS-induced RS, the cells were treated with hydrogen peroxide (H_2_O_2_). The knockdown of ORP3 or MAD2 was used reflect the different levels of severity of aneuploidy and potentially the different mechanisms of aneuploidy induction. While the knockdown of MAD2, a mitotic spindle assembly checkpoint factor, is known to result in severe aneuploidy [64,65,66], the knockdown or loss of ORP3, a member of the oxysterol-binding protein family and which is known to be involved in cell polarity and cell adhesion, induces low levels of aneuploidy by a yet unknown mechanism [15,67]. 

We found that γ-H2AX may be used for all three conditions, although pRPA2 is a more suitable marker for aneuploidy-induced replication stress. Moreover, the impact of telomerase on the replication stress level was addressed in the context of aneuploidy-induced RS, oncogene-induced RS, or ROS-induced replication stress. In line with previous reports, this study shows that telomerase activity reduces overall replication stress induced by aneuploidy-causing conditions, oncogenic mutations, or ROS.

## 2. Results

### 2.1. pRPA2 as Replication Stress Marker

Activated, i.e., phosphorylated Replication Protein A (RPA) binds single-stranded DNA (ssDNA) to prevent breaks during replication. More importantly, the phosphorylated 32 kDa subunit of RPA, pRPA2, is used as a marker of replication fork stalling, as the Cdc45-Mcm2-7-GINS (CMG) helicase further unwinds the DNA, but the forks are not further replicated [68].

#### 2.1.1. pRPA2 as Replication Stress Marker in Response to OI-RS 

The oncogenic mutant RAS, RAS^G12V^, was previously reported to induce replication stress [14,69]. Moreover, it was shown that telomerase can suppress oncogene replication stress and senescence [14]. We therefore ectopically expressed RAS^G12V^ in telomerase-negative BJ human fibroblasts and their telomerase-immortalized counterparts (BJ-hTERT) (see Section 4 and Supplementary Figure S1 for the generation of the cell transgenic lines).

**Figure 1:**
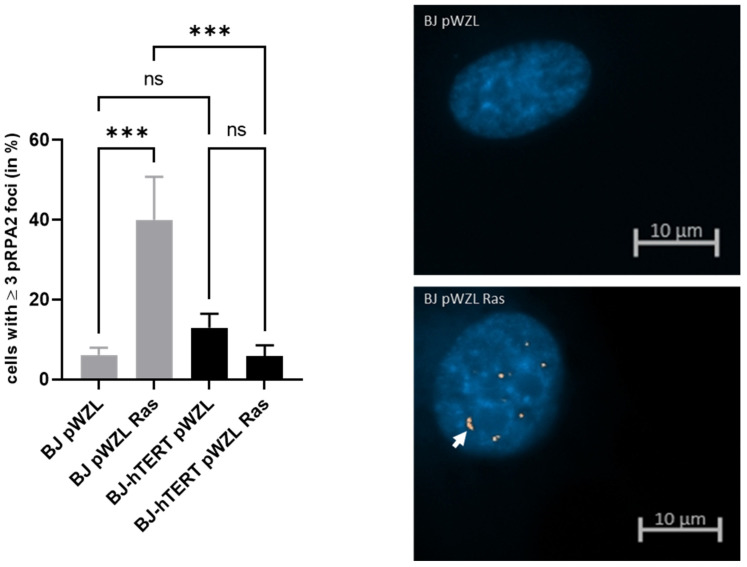
Evaluation of pRPA2 foci formation upon oncogene-induced replication stress. (**Left**) The bar plot displays the impact of ectopic H-RASG12V on pRPA2 foci formation in BJ and BJ-hTERT cells. pWZL represents the empty vector control. One-way ANOVA test was applied, followed by a Fisher’s LSD post hoc test, with a confidence interval of 95%. (**Right**) Representative images of BJ-pWZL (top) and BJ pWZL-RASG12V (bottom) are shown. A total of 210 cells were counted from *n* = 6 independent experiments. The representative pictures show DAPI-stained nuclei with nuclear pRPA2 foci (white arrow). ns (*p* > 0.05), *** (*p* ≤ 0.001).

The expression of RAS^G12V^ in BJ cells led to a marked (6.5-fold) and significant (*p* = 0.0006) increase of pRPA2 foci compared to the empty vector pWZL (~6.1% versus ~40%), indicating the accumulation of single-strand DNA upon oncogenic RAS^G12V^ signaling and this marker’s suitability for detecting RS (Figure 1). To address the impact of telomerase on OI-RS, pRPA2 foci were counted in BJ-RAS^G12V^ and BJ-hTERT-RAS^G12V^. Our data indicated that telomerase activation by the overexpression of hTERT results in a significant reduction of pRPA2 foci formation in response to OI-RS (Figure 1). While ≥3 pRPA2 foci were observed in only 5.87% of BJ-hTERT-RAS^G12V^ cells, about 40% of BJ- RAS^G12V^ cells contained ≥3 pRPA2 foci (*p* = 0.0006). On the other hand, we did not observe a significant difference in pRPA2 levels between BJ-pWZL and BJ-hTERT-pWZL (*p* = 0.4253). Moreover, unlike the BJ-pWZL/BJ-RAS^G12V^ comparison, the overexpression of RAS^G12V^ in BJ-hTERT cells did not result in an increased amount of pRPA2 foci (*p* = 0.4064) (Figure 1). 

In summary, the above data point out that pRPA2 is suitable for the detection of OI-RS. Moreover, telomerase activity suppresses OI-RS, similar to our previous observation showing that the activity of telomerase abrogates AI-RS [15].

#### 2.1.2. pRPA2 as Replication Stress Marker in Response to AI-RS 

Aneuploidy indicates any deviation from a normal chromosome number in a given species (euploidy) and is a hallmark of cancer [70]. Using a chromosome-combing assay (fiber assay) as well as pRPA2-, 53BP1-, and γ-H2AX staining, we have recently shown that aneuploidy-inducing gene knockdowns cause replication stress in telomerase-negative cells but not in telomerase-positive cells [15]. In the comparative experimental analyses in the present study, we knocked down two independent ploidy-control genes, MAD2L1 (referred to as MAD2), a spindle assembly checkpoint protein [64], and ORP3 (OSBP-related protein 3), a newly identified non-canonical ploidy-control protein, based on our previous observations [15]. 

**Figure 2:**
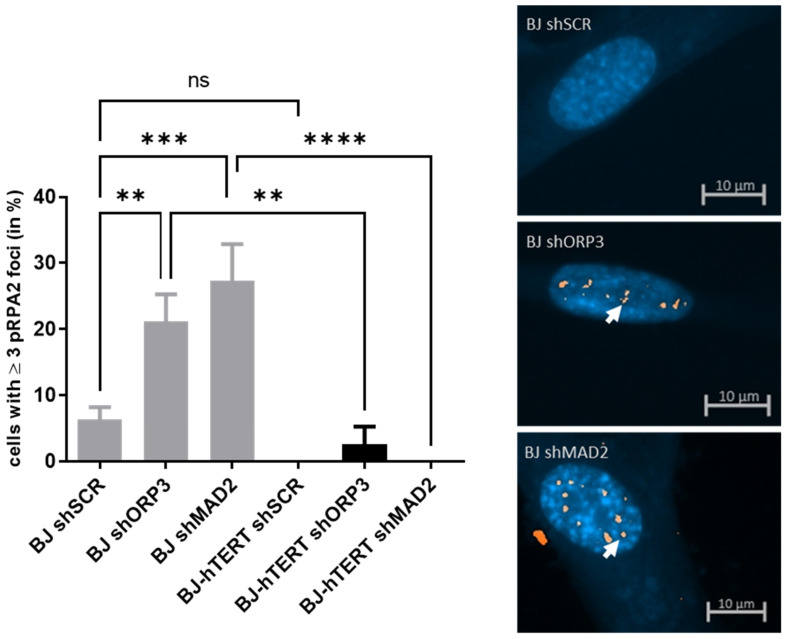
Evaluation of pRPA2 foci formation upon aneuploidy-induced replication stress. (**Left**) The bar plot displays the impact of ORP3 or MAD2 downregulation on pRPA2 foci formation in BJ and BJ-hTERT cells. pGIPZ-shSCR was used as an shRNA control vector. One-way ANOVA test was applied, followed by a Fisher’s LSD post hoc test, with a confidence interval of 95%. (**Right**) Representative images from BJ-shSCR (top), BJ-shORP3 (middle), and BJ-shMAD2 (bottom) cells. A total of 200 cells were counted from *n* = 4 independent experiments. The representative pictures show DAPI-stained nuclei with nuclear pRPA2 foci (white arrow). ns (*p* > 0.05), ** (*p* ≤ 0.01), *** (*p* ≤ 0.001), **** (*p* ≤ 0.0001).

The induction of AI-RS by the knockdown (KD) of ORP3 as well as MAD2 caused an increase of nuclear pRPA2-foci in BJ cells (Figure 2). While the KD of ORP3 resulted in more than a 3-fold increase in pRPA2 foci (~6.3% versus ~21%, *p* = 0.0064), MAD2 KD increased pRPA2 foci formation by more than 4-fold (~6.3% versus ~27.3%, *p* = 0.0004) in BJ cells. 

In line with our previous data, these results show that the KD of ORP3 and MAD2 induces replication stress in telomerase-negative human fibroblast BJ, and that the replication stress can be detected with pRPA2. 

The impact of telomerase on AI-RS was evaluated by pRPA2 foci formation in the telomerase-negative and telomerase-positive BJ fibroblasts. More importantly, both ORP3- as well as MAD2-KD did not significantly increase pRPA2 foci formation in telomerase-positive BJ-hTERT cells as compared to shSCR (*p* = 0.6343 and *p* > 0.9999, respectively). Furthermore, the comparison of BJ-shORP3 and BJ-hTERT-shORP3 revealed that the level of pRPA2 foci reduction is significant (*p* = 0.0024). Similarly, even more significant is the effect of telomerase in BJ-shMAD2 and BJ-hTERT-shMAD2 (*p* < 0.0001). Once again, these data are in line with our previous report showing that telomerase suppresses AI-RS (32). 

#### 2.1.3. pRPA2 as Replication Stress Marker in Response to ROS-Induced RS

Intracellular ROS, such as H_2_O_2_, can accumulate in response to a variety of agents, including oncogene activation or ionizing radiation [71,72], and cause replication stress [73]. Treatment of cells with H_2_O_2_ can mimic endogenous ROS accumulation and ROS-induced replication stress [74]. Recently, H_2_O_2_-treatment of human cells in the presence and absence of telomerase was used to evaluate the impact of telomerase on the amount and the localization of the DNA damage [16]. The authors used 53BP1 as a marker of genotoxic and oxidative stress in the persistence of DNA damage response during aging and cellular senescence. Here, we evaluated the suitability of pRPA2 for the detection of ROS-induced replication stress in the presence and absence of telomerase. 

We found that H_2_O_2_-treatment increases pRPA2 signals in BJ cells more than 8-fold. While only 4.5% of mock-treated cells show ≥3 pRPA2 foci per cell, this value increases to about 37% in the H_2_O_2_-treated BJ cells (*p* = 0.0027). This indicates that H_2_O_2_ treatment leads to an increase in ssDNA levels and thus to pRPA2 accumulation.

**Figure 3:**
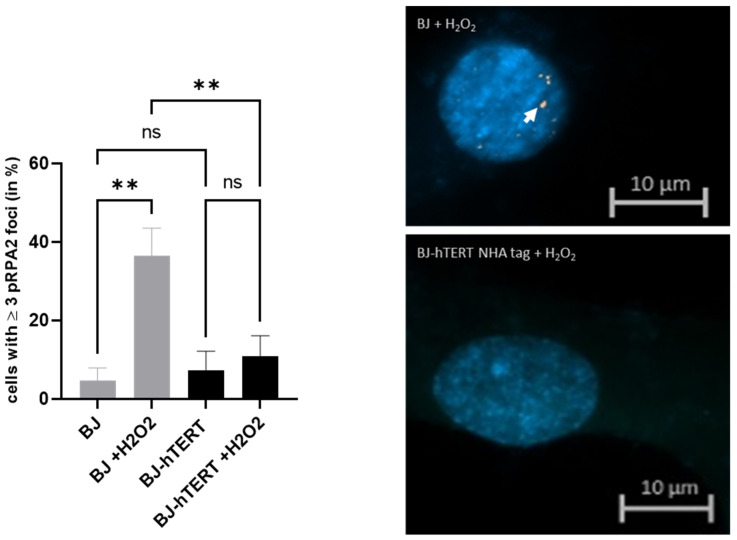
Evaluation of pRPA2 foci formation upon ROS-induced replication stress. (**Left**) The bar plot displays the impact of H_2_O_2_ treatment on pRPA2 foci formation in BJ and BJ-hTERT cells. One-way ANOVA test was applied, followed by a Fisher’s LSD post hoc test, with a confidence interval of 95%. (**Right**) Representative images from BJ (top) and BJ-hTERT (bottom) treated with H_2_O_2_. A total of 180 cells were counted from *n* = 3 independent experiments. The representative pictures show DAPI-stained nuclei with nuclear pRPA2 foci (white arrow). ns (*p* > 0.05), ** (*p* ≤ 0.01).

Unlike BJ cells, H_2_O_2_ treatment of BJ-hTERT did not lead to a significant increase of pRPA2 foci (*p* = 0.6460). More importantly, H_2_O_2_ treatment of BJ-hTERT cells induced significantly less pRPA2 foci as compared to BJ cells (*p* = 0.0065). Only ~10.85% of cells BJ-hTERT cells exhibited ≥3 pRPA2 foci as compared to ~36.7% BJ cells showing ≥3 pRPA2 foci (Figure 3).

### 2.2. γ-H2AX as Replication Stress Marker

γ-H2AX is a marker for DNA damage response (DDR) and is activated upon double-strand breaks (DSB). Frequently, γ-H2AX is used to evaluate DNA replication stress as persistent RFS results in DSB and the activation of γ-H2AX. 

#### 2.2.1. γ-H2AX as Replication Stress Marker in Response to OI-RS

**Figure 4:**
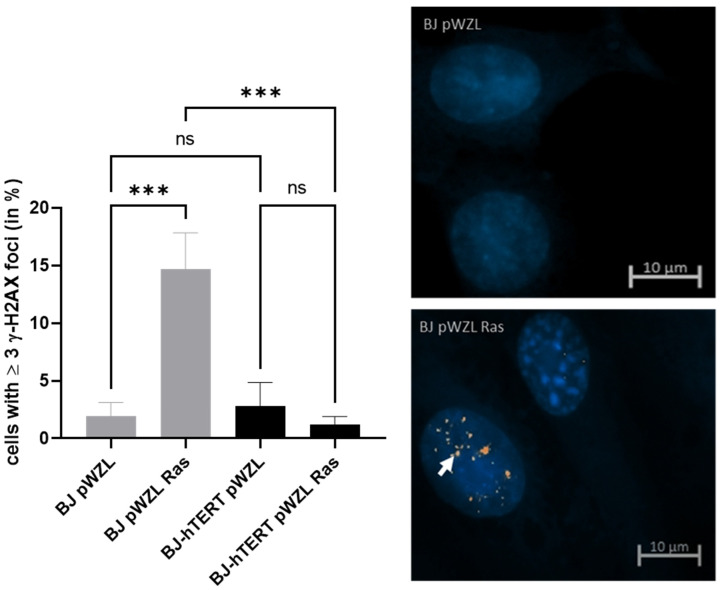
Evaluation of γ-H2AX foci formation upon oncogene-induced replication stress. (**Left**) The bar plot displays the impact of ectopic H-RAS^G12V^ on γ-H2AX foci formation in BJ and BJ-hTERT cells. pWZL represents the empty vector control. One-way ANOVA test was applied, followed by a Fisher’s LSD post hoc test, with a confidence interval of 95%. (**Right**) Representative images of BJ-pWZL (top) and BJ pWZL-RAS (bottom). A total of 200 cells were counted from *n* = 4 independent experiments. The representative pictures show DAPI stained nuclei with nuclear pRPA2 foci (white arrow). ns (*p* > 0.05), *** (*p* ≤ 0.001).

The overexpression of RAS^G12V^ increases the level of γ-H2AX foci in BJ cells as compared to the control cells (BJ-PWZL) about 8-fold (~1.9% versus ~14.66%; *p* = 0.0007) (Figure 4). This points out that the replication stress induced by oncogene overexpression can be readily detected by γ-H2AX. More importantly, the determination of OI-RS by γ-H2AX provides a better marker compared to pRPA2; the replication stress is clearer and does not exhibit as much background as with pRPA2. The replication stress increase detected by γ-H2AX was 8-fold (~1.9% versus ~14.66%) as compared to the 6.5-fold increase by pRPA2. The reduction from BJ-RAS to BJ-hTERT-RAS is also clearer in the evaluation with γ-H2AX. Telomerase activity reduces the replication stress by ~12.3% via γ-H2AX evaluation, but only by ~6.8% in the evaluation via pRPA2. 

Similarly, the suppression of OI-RS by telomerase cells can clearly be observed when γ-H2AX is used as a marker (*p* = 0.0005). While 14.66% of BJ-RAS^G12V^ cells show ≥3 γ-H2AX foci, only 1.1% of BJ-hTERT-RAS^G12V^ cells exhibit ≥3 γ-H2AX foci, indicating about 13-fold less γ-H2AX foci formation in the presence of telomerase (Figure 4). In fact, the number of BJ-hTERT-RAS^G12V^ cells displaying ≥3 γ-H2AX foci is comparable to that of BJ-hTERT-pWZL (~1.1% versus ~2.7%; *p* = 0.5803). 

#### 2.2.2. γ-H2AX as Replication Stress Marker in Response to AI-RS

We observed a significant increase in γ-H2AX foci formation upon aneuploidy induction through the KD of ORP3 (~8.5% versus ~32.1%; *p* = 0.0015) and upon the KD of MAD2 (~8.5% versus ~57.9%; *p* < 0.0001). The highly significant *p*-values indicate that γ-H2AX is a reliable choice of marker for the evaluation of aneuploidy-induced replication stress. 

**Figure 5:**
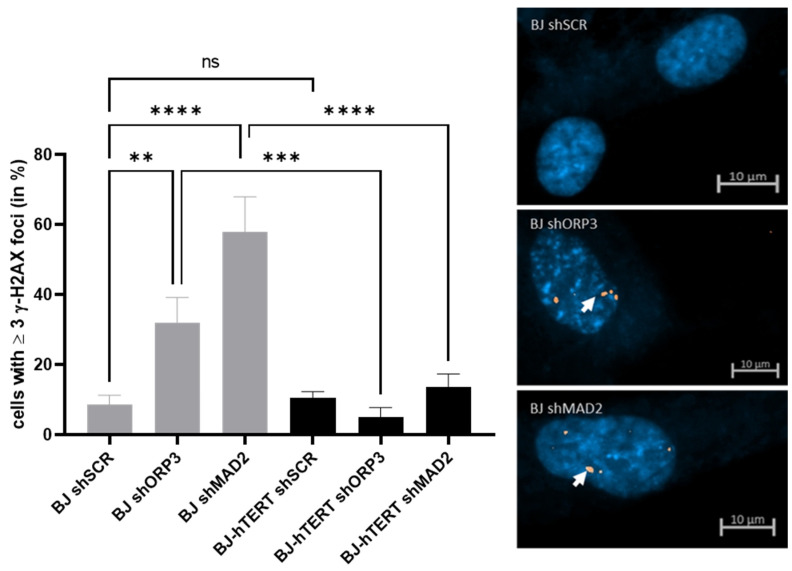
Evaluation of γ-H2AX foci formation upon aneuploidy-induced replication stress. (**Left**) The bar plot displays the impact of ORP3 or MAD2 downregulation on γ-H2AX foci formation in BJ and BJ-hTERT cells. pGIPZ-shSCR was used as an shRNA control vector. One-way ANOVA test was applied, followed by a Fisher’s LSD post hoc test, with a confidence interval of 95%. (**Right**) Representative images from BJ-shSCR (top), BJ-shORP3 (middle), and BJ-shMAD2 (bottom) cells. A total of 210 cells were counted from *n* = 6 independent experiments. The representative pictures show DAPI-stained nuclei with nuclear pRPA2 foci (white arrow). ns (*p* > 0.05), ** (*p* ≤ 0.01), *** (*p* ≤ 0.001), **** (*p* ≤ 0.0001).

In BJ-hTERT, γ-H2AX foci formation is significantly suppressed upon ORP3 or MAD2 KD as compared to their KD in BJ cells (Figure 5). The KD of ORP3 induced 6-fold less γ-H2AX foci in BJ-hTERT as compared to BJ cells (~5% versus ~32%; *p* = 0.0004), and the KD of MAD2 induced 4-fold less γ-H2AX foci (~13.7% versus ~57.9%; *p* < 0.0001).

#### 2.2.3. γ-H2AX as Replication Stress Marker in Response to ROS-Induced RS

**Figure 6:**
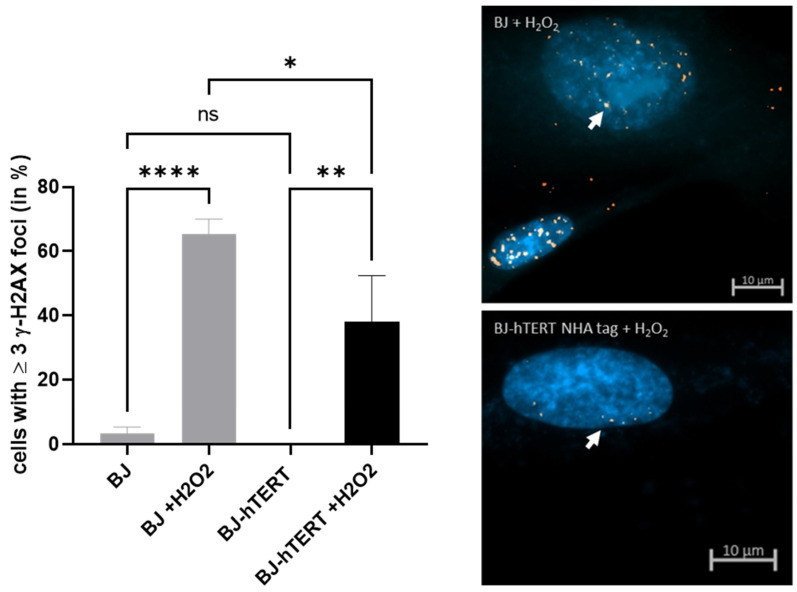
Evaluation of γ-H2AX foci formation upon ROS-induced replication stress. (**Left**) The bar plot displays the impact of H_2_O_2_ treatment on γ-H2AX foci formation in BJ and BJ-hTERT cells. One-way ANOVA test was applied, followed by a Fisher’s LSD post hoc test, with a confidence interval of 95%. (**Right**) Representative images from BJ (top) and BJ-hTERT (bottom) treated with H_2_O_2_. A total of 160 cells were counted from *n* = 4 independent experiments. The representative pictures show DAPI-stained nuclei with nuclear pRPA2 foci (white arrow). ns (*p* > 0.05), * (*p* ≤ 0.05), ** (*p* ≤ 0.01), **** (*p* ≤ 0.0001).

We observed a nearly 19-fold increase in γ-H2AX foci formation in BJ cells upon H_2_O_2_ treatment (~3.3% versus ~65%; *p* < 0.0001), indicating that these cells strongly suffer from DNA damage under the treatment conditions (Figure 6). Moreover, we observed a significant but faint suppression of γ-H2AX foci formation in the presence of telomerase upon H_2_O_2_ treatment (*p* = 0.0118), indicating that this molecular marker is less suitable for the evaluation of the impact of telomerase in the context of ROS-induced RS (Figure 6). In fact, a significant amount of the H_2_O_2-_treated BJ-hTERT cells exhibited ≥3 γ-H2AX foci, while untreated BJ-hTERT cells were completely negative for this marker (~38.1% versus 0%; *p* = 0.0014). 

### 2.3. BP1 as Replication Stress Marker

Tumor suppressor p53-binding protein 1 is a key player in double-strand break (DSB) repair [39]. Depending on the stage of the cell cycle, 53BP1 promotes different repair pathways: during G1, it promotes non-homologous end joining (NHEJ), whereas in the S and G2 phases, 53BP1 is involved in DNA end resection and the homologous recombination of DSB. The decision with regard to which pathway the DSBs are to be repaired is mediated through the interaction partner of 53BP1 [39]. 

Though 53BP1 was recently used to detect and evaluate fragile telomeres and telomere dysfunction-induced foci [14,16], this marker was not directly tested as a general marker for replication stress.

#### 2.3.1. 53BP1 as Replication Stress Marker in Response to OI-RS 

**Figure 7:**
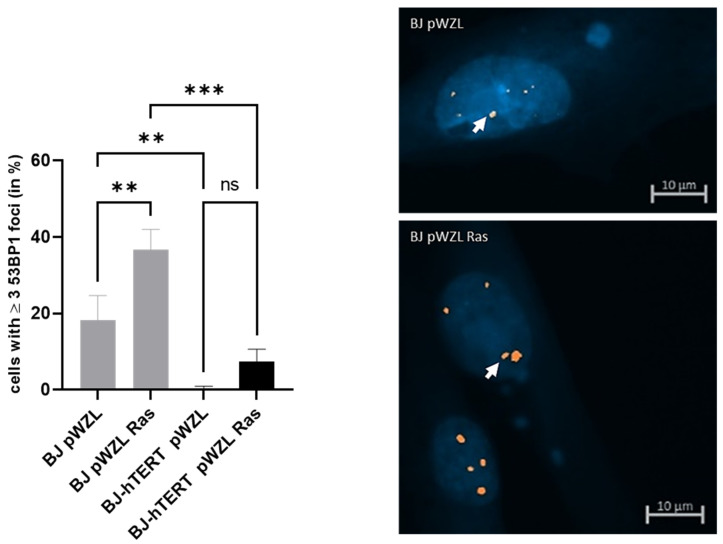
Evaluation of 53BP1 foci formation upon oncogene-induced replication stress. (**Left**) The bar plot displays the impact of ectopic H-RAS^G12V^ on 53BP1 foci formation in BJ and BJ-hTERT cells. pWZL represents the empty vector control. One-way ANOVA test was applied, followed by a Fisher’s LSD post hoc test, with a confidence interval of 95%. (**Right**) Representative images of BJ-pWZL (top) and BJ pWZL-RAS (bottom). A total of 200 cells were counted from *n* = 5 independent experiments. The representative pictures show DAPI-stained nuclei with nuclear pRPA2 foci (white arrow). ns (*p* > 0.05), ** (*p* ≤ 0.01), *** (*p* ≤ 0.001).

The expression of RAS^G12V^ in BJ cells increases the proportion of cells displaying ≥3 53BP1 foci as compared to BJ-pWZL with only a 2-fold increase (~18.2% versus 36.6%) (Figure 7). However, this difference is less significant (*p* = 0.0083), likely due to background 53BP1 signals in a high proportion of BJ-PWZL cells (about 20%). Based on this data, 53BP1 is less suitable for the evaluation of OI-RS. Interestingly, however, we found that the proportion of cells with 53BP1 signals is reduced in the presence of telomerase in the context of OI-RS (~36.6% in BJ-RAS^G12V^ compared to ~7.3% in BJ-hTERT-RAS^G12V^; *p* = 0.0002).

#### 2.3.2. 53BP1 as Replication Stress Marker in Response to AI-RS

**Figure 8:**
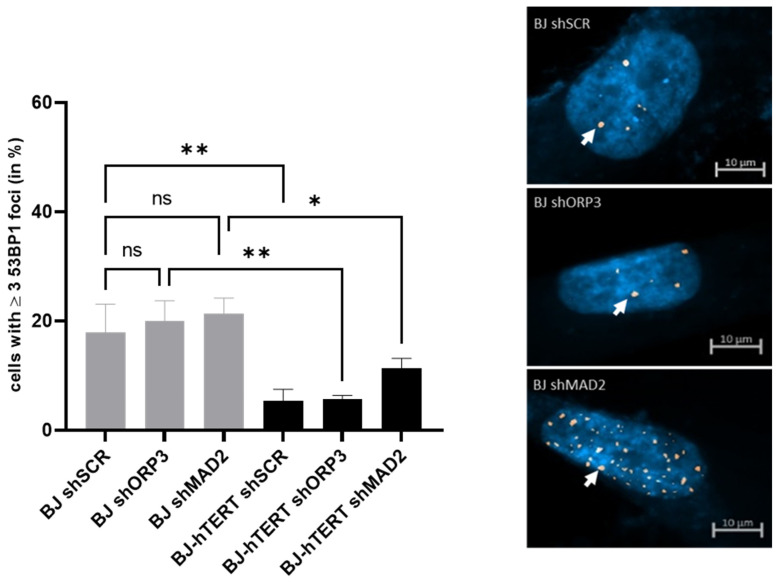
Evaluation of 53BP1 foci formation upon aneuploidy-induced replication stress. (**Left**) The bar plot displays the impact of ORP3 or MAD2 downregulation on 53BP1 foci formation in BJ and BJ-hTERT cells. pGIPZ-shSCR was used as an shRNA control vector. One-way ANOVA test was applied, followed by a Fisher’s LSD post hoc test, with a confidence interval of 95%. (**Right**) Representative images from BJ-shSCR (top), BJ-shORP3 (middle), and BJ-shMAD2 (bottom) cells. A total of 300 cells were counted from *n* = 6 independent experiments. The representative pictures show DAPI-stained nuclei with nuclear pRPA2 foci (white arrow). ns (*p* > 0.05), * (*p* ≤ 0.05), ** (*p* ≤ 0.01).

The evaluation of the aneuploidy induction by the shRNA-mediated KD of ORP3 or MAD2 revealed that there is no significant increase in 53BP1 foci formation compared to shSCR (*p* = 0.6283 or *p* = 0.4240, respectively) (Figure 8). These results point out that 53BP1 is not a suitable marker for the detection and evaluation of AI-RS. 

Interestingly, telomerase activity reduces the proportion of cells displaying ≥3 53BP1 foci upon ORP3 KD (~20.04% versus ~5.6%; *p* = 0.0023) and upon MAD2 KD (~21.4% versus ~11.3%; *p* = 0.0270) (Figure 8). This points out that even though the increase in BJ cells upon aneuploidy induction is not significant, the abrogation upon the introduction of telomerase is still significant.

The increase of 53BP1 foci upon ORP3 KD and MAD2 KD in BJ-hTERT cells remains insignificant (~5.4% versus ~5.6%; *p* = 0.9636, and ~5.4% versus ~11.3%; *p* = 0.1806, respectively). 

#### 2.3.3. 53BP1 as Replication Stress Marker in Response to ROS-Induced-RS

**Figure 9:**
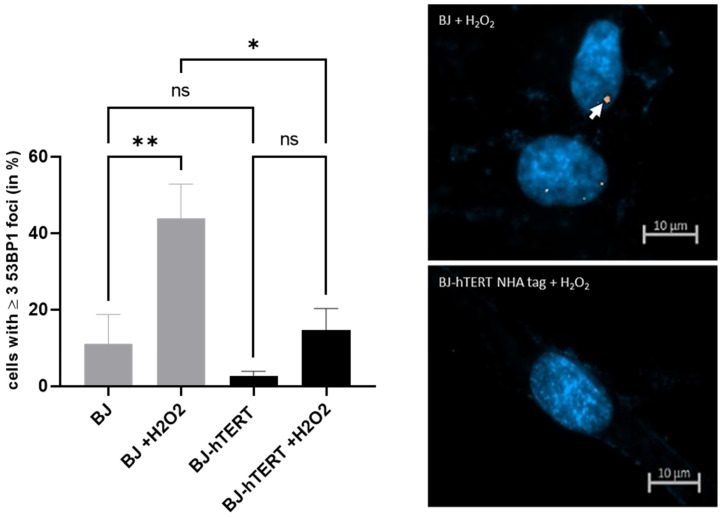
Evaluation of 53BP1 foci formation upon ROS-induced replication stress. (**Left**) The bar plot displays the impact of H_2_O_2_ treatment on 53BP1 foci formation in BJ and BJ-hTERT cells. One-way ANOVA test was applied, followed by a Fisher’s LSD post hoc test, with a confidence interval of 95%. (**Right**) Representative images from BJ (top) and BJ-hTERT (bottom) treated with H_2_O_2_. A total of 170 cells were counted from *n* = 4 independent experiments. The representative pictures show DAPI-stained nuclei with nuclear pRPA2 foci (white arrow). ns (*p* > 0.05), * (*p* ≤ 0.05), ** (*p* ≤ 0.01).

We observed a noticeable and significant increase in 53BP1 signals in BJ cells upon H_2_O_2_ treatment (~43.7% versus ~11.05%; *p* = 0.0063) (Figure 9). This result indicates that 53BP1 is a suitable marker for ROS-induced RS. We also observed that telomerase activity suppresses ROS-induced 53BP1 foci as compared to telomerase negative cells (~43.7% versus ~14.6%; *p* = 0.0121), though the 53BP1 levels in H_2_O_2_-treated BJ-hTERT cells are slightly higher as compared to untreated BJ-hTERT cells (~2.5% versus ~14.6%; *p* = 0.2728) (Figure 9). Another observation is that the proportion of cells with 53BP1 foci is lower for aneuploidy-induced replication stress and for ROS formation as compared to the proportion of cells with γ-H2AX foci, whereas the proportion of cells with 53BP1 foci is higher than those with γ-H2AX foci for OI-RS. 

### 2.4. Impact of ATM and ATR Inhibitors on pRPA2 Foci Accumulation

The above data rely on IF staining, which is the preferred detection method of RS-induced foci formation and DNA damage pathway activation. As an attempt to see if the accumulation of RS can also be detected by the immunoblot method, we performed initial WB experiments in the context of H_2_O_2_-treatment. The results of this experiment show that Western blot may also be used to observe the induction of marker proteins in response to RS (Supplementary Figure S2). Interestingly, however, we observed several pRPA2-specific bands in the WB. Moreover, while the intensity of the larger protein band was reduced in the H_2_O_2_-treated cells, the intensity of the smaller protein band was slightly increased (Supplementary Figure S2). Considering that the antibody specifically detects Ser33-pRPA2, one may assume further modification of the Ser33-pRPA2 upon H_2_O_2_-treatment. Further experiments are required to clarify this aspect. 

**Figure 10:**
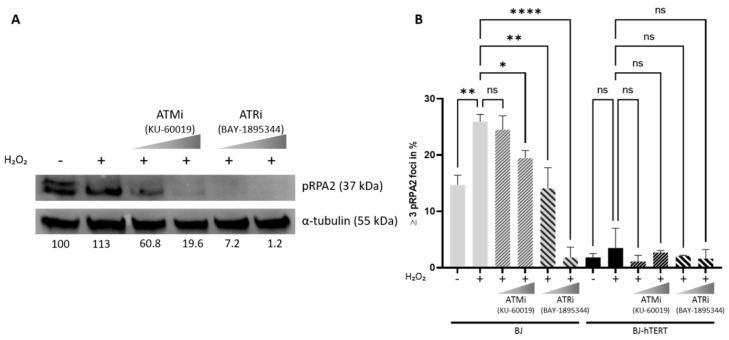
Inhibition of ATM or ATR impairs ROS-induced replication stress in BJ cells. BJ and BJ-hTERT cells were subjected to the inhibitors KU-60019, an ATM kinase inhibitor (1–3 µM), and BAY-1895344, an ATR inhibitor (5–25 nM) for 1 h, before the addition of 400µM H_2_O_2_ for another hour. (**A**) A representative Western Blot showing the decrease of pRPA2 levels upon treatment with ATM and ATR inhibitors in BJ cells. As a loading control, α-tubulin is shown. Intensity ratios of the pRPA2 lower band normalized to α-tubulin, and relative to BJ cells without treatment, are indicated below the blot (in %). (**B**) The bar plot evaluates the impact of H _2_O_2_ on pRPA2 foci formation after ATM and ATR inhibition in BJ and BJ-hTERT cells. One-way ANOVA test was applied, followed by a Fisher’s LSD post hoc test, with a confidence interval of 95%. A total of 310 cells were counted from *n* = 2 independent experiments. ns (*p* > 0.05), * (*p* ≤ 0.05), ** (*p* ≤ 0.01), **** (*p* ≤ 0.0001).

As ATM and the ATR pathway have been shown to mediate RS and DNA damage induction, we performed Western blot (WB) and IF experiments in the absence and presence of ATM and ATR inhibitors. Based on our above presented results showing that pRPA2 is a suitable and reliable marker for the detection and quantification of RS, we focused on this marker in combination with H_2_O_2_-treatment for a proof-of-principle experiment in this context. The results show that both ATM and ATR inhibition impair the accumulation of pRPA2 foci in response to H_2_O_2_-treatment, while neither ATM nor ATR inhibition interferes with pRPA2 reduction in the presence of telomerase (Figure 10A). Worthy of note is the fact that we could again observe a reduction in the signal intensity of the upper pRPA2 band (Figure 10A, first lane: untreated control) and the increased signal intensity of the lower band in H_2_O_2_-treated cells (Figure 10A, second lane). Nevertheless, both bands fade in the cells with ATM- or ATR-inhibitors (Figure 10A, lanes 3–6). More importantly, with respect to RS-induction and the impact of telomerase on the alleviation of pRPA2 accumulation, the results of the Western blot correspond to the results obtained by IF (Figure 10B).

## 3. Discussion

Faithful replication is essential for maintaining genome integrity and for the transfer of intact genomic material to the progeny. Defects in replication can result in an accumulation of mutations, lead to genome instability, and promote the tumorigenic conversion of multicellular organisms. There is mounting evidence that replication stress can cause genome instability and promote cancer [26,75,76,77]. Cells with an intact DNA damage checkpoint respond to replication stress to prevent genome instability [78,79]. ATR- and ATM-mediated DDR are central to the sensing of replication stress and to the prevention of genome instability through the activation of downstream factors, including pRPA2, γ-H2AX, and 53BP1 [13,17,40,80,81,82,83]. 

Recent studies, including our own results, utilized p-RPA2-, γ-H2AX-, or 53BP1-staining to mark cells with replication stress that had been induced by aneuploidy (AI-RS), oncogenic mutations (OI-RS), and reactive oxygen species (ROS-induced RS), and to quantify the level of replication stress in these cells [14,15,16]. Since a direct comparative study that assessed the suitability of these RS-markers under different RS-inducing conditions is lacking, we addressed this question in the present study. Moreover, the impact of telomerase on the level of replication stress formation in the context of the different markers was evaluated. Based on our previous investigations, we used the telomerase-negative BJ fibroblasts and their telomerase-positive derivatives (BJ-hTERT) to induce RS, either by the overexpression of an active oncogenic RAS variant (RAS^G12V^: for OI-RS), by the knockdown of *ORP3* or *MAD2* (for AI-RS), or by the treatment with hydrogen peroxide (H_2_O_2_) as a source of ROS, respectively. 

The evaluation of the RS marker proteins revealed differences in their suitability and reliability as potential markers in response to RS triggered by the different conditions (AI-RS, OI-RS, and ROS-induced RS, respectively). We found that both γ-H2AX and pRPA2 may be used as an RS marker for all three RS-inducing conditions in telomerase-negative BJ cells, noticing that γ-H2AX provides lower background signals in the control BJ cells. On the other hand, 53BP1 is only suitable for the evaluation of ROS-induced RS under the experimental conditions used here. However, pRPA2 staining provides more significant results and seems to be more reliable for the evaluation of ROS-induced RS as compared to 53BP1 or γ-H2AX. Therefore, pRPA2 may be the marker of choice for the assessment of ROS-induced RS. Although γ-H2AX foci formation is strongly and significantly induced upon H_2_O_2_-treatment and may be considered for the evaluation of ROS-induced RS in the telomerase-negative background, it performs less significantly in the context of the telomerase-mediated reduction of RS in BJ-hTERT cells (the proportion of γ-H2AX foci-containing cells upon H_2_O_2_-treatment is ~65% for BJ and ~38.1% for BJ-hTERT, while the *p*-value is *p* = 0.0118). Interestingly, Suram et al. [14] used 53BP1 to evaluate RS induced by hydroxyurea (HU) and aphidicolin response in BJ and BJ-hTERT cells. Although only telomeric foci formation was determined in this study, the authors observed that RS-triggered 53BP1 foci formation was alleviated in the presence of TERT, i.e., telomerase. Moreover, the authors documented both non-telomeric and telomeric foci formation in both the absence and presence of telomerase in response to active RAS overexpression. In addition, they observed that non-telomeric 53BP1 foci signals decrease in the presence of telomerase. Worthy of note is the fact that despite using the same experimental setting and yielding the same results as Suram and colleagues, our findings indicate 53BP1 as less suitable for the evaluation of OI-RS. Hewitt et al. [16] involved γ-H2AX and 53BP1 as markers for characterizing the DDR activity of non-telomeric (non-TAF) and telomere-associated foci formation (TAFs) in response to X-Ray and H_2_O_2_-treatment in telomerase-negative MRC5 fibroblasts and their immortal counterparts, MRC5-hTERT [16]. The authors demonstrated that irradiation induces γ-H2AX-TAFs and non-TAFs in MRC5 fibroblasts, but only non-TAFs were reduced in MRC5-hTERT. Similarly, when evaluating 53BP1-TAFs and non-TAFs in mouse embryonic fibroblasts (MEFs) in response to H_2_O_2_-treatment, the authors observed a reduction of non-TAFs in the telomerase-positive MEFs. In line with their data, we were also able to document a suppression of γ-H2AX- and 53BP1-non-TAF signals in the presence of telomerase despite the lack of X-Ray regimen in our experimental setting. Whether irradiation-triggered non-TAF formation is reduced in MRC5 vs. MRC5-hTERT cells and in telomerase-negative vs. telomerase-positive MEFs upon employing pRPA2 as a marker remains to be elucidated. As we did not address telomere-associated foci formation in this study, we cannot compare our results to TAF data in the study by Hewitt et al. [16]. However, in a previous report [15], our fiber assay provided direct evidence for AI-RS and the induction of TAFs in the absence of telomerase. At the same time, the telomerase activity alleviated TAF formation, suggesting that pRPA2 might be more suitable for TAF evaluation under all RS-inducing conditions.

Taken together, this study extends our previous findings and shows that telomerase activity reduces overall replication stress caused by aneuploidy-causing conditions, oncogenic mutations, or ROS and defines pRPA2 as the most suitable marker for the evaluation of the telomerase-dependent alleviation of RS in all three conditions. Particularly noteworthy is that these conclusions are based on the evaluation of the IF staining results and have proven to be consistent and quantifiable. Preliminary data by Western blot experiments for the H_2_O_2_ treatment support the results of IF quantifications, showing the induction of all three markers upon H_2_O_2_ treatment in BJ cells, while we observe their reduction in BJ-hTERT cells, though to a lower level for γ-H2AX. This observation suggests that WB could also be a choice for the evaluation of RS-induced accumulation of the marker proteins and the impact of telomerase, although further confirmation for all conditions is necessary (Supplementary Figure S2).

More importantly, the IF staining involving one of these markers represents a reliable alternative tool to the fiber assay which was used to detect RS at telomeres by Meena et al. [15] and Suram et al. [14]. The advantages of the fluorescence staining for the evaluation of RS by these marker proteins are the easy experimental setting and fast read-out of the results in comparison to the time-consuming optimization and the high technical difficulty of the fiber assay. Finally, in addition to being used as markers for experimental studies, the markers presented in this study could be useful in determining the accumulation of cells with impaired proliferation or in accumulating DNA damage during aging.

We wish to emphasize here that although it was not the main purpose of this study to decipher how telomerase suppresses replication stress, based on published data, we speculated that telomerase modulates ATM- and ATR-signaling pathways in response to conditions that induce genome instability and replication stress [84,85,86]. While the telomere-related connection of ATM-ATR and telomerase is well-documented, we are not aware of any report showing the direct impact of telomerase on the ATM- and ATR-signaling cascades; telomere dysfunction activates the ATM-ATR-dependent DDR, and it was demonstrated that ATM- and ATR-signaling regulate the recruitment of telomerase to telomeres (e.g., [84,87]). Moreover, telomerase repairs collapsed replication forks at telomeres [86]. However, whether telomerase impacts ATM-ATR signaling in the telomere-length-independent context is not clear. Our data demonstrate that the presence of telomerase impairs the activation of RS/DDR-induced foci formation, as measured by the activation of the primary target proteins of ATM and the ATR-signaling cascade, pRPA2 and γ-H2AX, respectively. The results of our proof-of-principle experiments (Figure 10) show that inhibition of both kinases impairs the accumulation of RS in telomerase-negative cells, indicating that the induction of RS is prevented by telomerase through the suppression of ATM- and ATR-kinase activation. However, our data do not exclude the fact that the telomerase-meditated reduction of pRPA2 foci numbers and the pRPA2 foci number reduction in response to ATM- or ATR-inhibition are mediated by independent pathways. This function of telomerase may be beneficial in cells with intact checkpoints but could allow the continuous proliferation of damaged cells or cells with impaired replication and promote cancer formation. Although telomerase acts primarily at the telomeric regions, in summary, the data of this study together with previous reports indicate that the new function of telomerase, that of suppressing RS and DNA-damage, is not only restricted to telomeric regions but may also act in non-telomeric regions. For experimental simplicity, the initial studies in this report were performed without the telomeric co-staining approach. Nevertheless, the results clearly emphasize the impact of telomerase in alleviating AI-RS, OI-RS, and ROS-induced RS. As RS can promote genome instability, these data support the idea that the downregulation of telomerase in human cells acts as a tumor suppressor mechanism as the cells with aneuploidy-inducing or oncogenic mutations induce senescence or apoptosis. In particular, we observed that RAS^G12V^ expression and MAD2 knockdown induced a rapid growth arrest both in BJ and BJ-hTERT cells. More importantly, while BJ cells remained in permanent growth arrest, BJ-hTERT cells were rescued from the initial growth arrest and continued to proliferate (data not shown). This new function of telomerase is independent of its role in telomere length regulation. Particularly noteworthy is the fact that TERT was shown to modulate the chromatin state and DNA damage responses, including the p53-dependent pathway [50,52]. It was also shown that telomerase may localize to mitochondria and protect cells from nuclear as well as mitochondrial DNA from damage [49,53]. Although we could not observe the extra-nuclear localization of Flag-hTERT in our experiments (unpublished observations), more in-depth studies are required to elaborate the mechanisms of how telomerase suppresses RS in normal and tumor cells. 

Finally, considering that telomerase activity is absent in the vast majority of human somatic cells, the activation of RS in response to harmful genetic alterations in these cells may be considered as a tumor suppression mechanism. On the other hand, telomerase activity is detectable in the majority of human tumors, in line with the idea that telomerase can support tumor formation, not only by stabilizing telomere functionality but also by the suppression of RS in cells with harmful mutations, thus allowing the survival of genomically instable cells [88,89]. 

## 4. Material and Methods

### 4.1. Cell Lines and Generation of Transgenic Cells

BJ cells were purchased from ATCC (ATCC LGC Standards GmbH, Manassa, Virginia, USA; Cat.-Nr. CRL-2522). BJ-hTERT cells were generated by transduction with the pWZL-Blast-Flag-HA-hTERT retroviral vector (Addgene; Cat.-Nr. 22396). All cells were cultured in DMEM high glucose, penicillin (10.000 unit/mL), streptomycin (10 mg/mL), and 10% FCS at 37 °C and 5% CO_2_. In order to knock down the *ORP3* and *MAD2* genes, we used shRNA constructs along with a scrambled construct, as previously described [15,67]. Efficient knockdown of both proteins could be achieved, as indicated by the Western blot experiments (Supplementary Figure S1A,B, respectively). Similarly, the Western blot result confirms the ectopic expression of the oncogenic RAS protein (RAS^G12V^) (Supplementary Figure S1C). The activity of telomerase due the expression of the ectopic TERT protein was confirmed by the Telomerase Repeat Amplification Protocol (TRAP)-Assay (Supplementary Figure S1D). For this purpose, we used nuclear lysates of BJ and BJ-hTERT cells (BJ-hTERT cells express the human telomerase reverse transcriptase protein with an N-terminal Flag-tag. In the following, BJ-hTERT and BJ-FLAG-hTERT are used synonymously), followed by immunoprecipitation (IP) with an anti-Flag antibody. The IP-lysates were then used to determine the activity of telomerase by the TRAP assay (Supplementary Figure S1D) in BJ-hTERT cells.

### 4.2. Plasmids

The pGIPZ plasmids used to generate the shRNA-mediated gene knockdown were as described in [15]. The following plasmids were obtained from Addgene: pWZL-Blast-Flag-HA-hTERT, a gift from William Hahn (Addgene plasmid #22396; http://n2t.net/addgene (accessed on 20 October 2011): 22396; RRID:Addgene_#22396), pWZL-hygro empty and pWZL-hygro-RAS^G12V^ vectors, a gift from Scott Lowe (Addgene plasmids #18750 and #18749). 

### 4.3. Antibodies

Following antibodies were used: anti-ORP3 (Santa Cruz Biotechnology, Dallas, TX, USA; Cat.-Nr. sc-398326, WB: 1:1000), anti-MAD2 (Bethyl Laboratories, Montgomery, TX, USA; Cat.-Nr. A300-301A WB: 1:1000), anti-H-RAS (NSJ Bioreagents, San Diego, Ca, USA; Cat.-Nr. F41616 WB: 1:1000), anti-phospho-H2AX (Ser139) (Merck Millipore, Burlington, MA, USA; Cat.-Nr. 05-636; WB and IF 1:500), anti-53BP1 (Novus biologicals Littleton, CO, USA; Cat.-Nr. N100-304; WB: 1:100 and IF 1:500), anti-phospho-RPA2 (Ser33) (Bethyl Laboratories, Montgomery, TX, USA; Cat.-Nr. A300-246A; WB: 1:1000 and IF 1:300), anti-FLAG (Sigma Aldrich, St. Louis, MO, USA; Cat.-Nr. F1804, IF: 1:100), anti-α-tubulin (Sigma Aldrich, St. Louis, MO, USA; Cat.-Nr. T6199; WB 1:1000), and anti-ß-actin (Sigma Aldrich, St. Louis, MO, USA; Cat.-Nr. A1978; WB 1:5000). The expected size of the proteins is indicated in kDa for each figure.

### 4.4. Nuclear Extract Preparation 

BJ and BJ-hTERT cells were seeded on 15 cm^2^ plates and were collected after 48 h at a confluency of ~70%. The cells were washed in 1 × PBS and scraped off the plates with 1 mL of PBS. The nuclear extracts were prepared according to the Dignam protocol [90]. Nuclear extracts were transferred into a dialysis button and put into AM100 (100 mM KCl, 20 mM Tris pH = 8, 5 mM MgCl_2_, 0.2 mM EDTA10% Glycerol, and 1 mM DTT). The samples were dialyzed for 4–6 h at 4 °C in AM100 and then stored at −80 °C until use.

### 4.5. Immunoprecipitation (IP) and IP-TRAP

A total of 300 µg nuclear extract of BJ and BJ-hTERT cells were incubated with 25 µL Protein-A Agarose IgG beads (Thermo Scientific, Waltham, MA, USA; Pierce, Cat.-Nr. 20334) or 25 µL Protein-A Agarose Flag beads (Sigma Aldrich; Cat.-Nr. A2220) in a total volume of 150 µL of AM100 supplemented with complete protease inhibitor (Hoffmann-La Roche, Basel, Switzerland; Cat.-Nr. 11687598001). The samples were incubated with the antibodies and beads for binding at 4 °C for 2.5 h under rotation and washed 3 times with 300 µL of AM150 containing 0.1% NP40. For the TRAP assay, the beads were resuspended in 25 µL CHAPS lysis buffer (Merck Millipore, Burlington, MA, USA; Cat.-Nr. S7705), and 2 µL were used. IP-TRAP was performed, as described in [62].

### 4.6. TRAP Assay 

TRAP assay was performed, as previously described [91].

### 4.7. Western Blot

Whole-cell lysates were prepared by scraping the cells with RIPA buffer and mechanical disruption. The lysates were centrifuged for 30 min at 14,000 rpm at 4 °C. Samples were run on a 10% SDS-PAA gel at 200 volts, and proteins were transferred onto the PVDF membrane at 4 °C. The membrane was blocked in 5% milk for 1 h and incubated with the first antibody overnight at 4 °C. Subsequent washes were performed with 0.1% Tween-20 in TBS.

### 4.8. Immunofluorescence

The cells were seeded in 6-well plates on cover slips for 36 h. The cells were fixed with 4% FA, washed with PBS, permeabilized with 1% Triton-x-100 in PBS, washed 3 times with PBS, and blocked in 1% BSA in PBS. Overnight antibody incubation at room temperature was followed by 3 washes with 0.1% Tween20-PBS and were incubated for 1 h with the appropriate Cy-3-labelled secondary antibody (Sigma Aldrich, St. Louis, MO, USA; Cat.-Nr. C2181; and Jackson ImmunoResearch, West Grove, PA, USA; Cat.-Nr. 111-165-006; 1:1000). The coverslips were washed 3 times with 0.1% Tween20-PBS before mounting with antifade containing DAPI (Life technologies, Carlsbad, CA, USA; Cat.-Nr. P36935).

### 4.9. Induction of ROS

To induce ROS, the cells were treated with 400 µM H_2_O_2_ in a pure DMEM medium at 37 °C for 1 h. Pure DMEM was used as a negative control.

### 4.10. Microscopy

The analyses of the immunofluorescence staining were carried out on the Zeiss system Axio Imager. M2 with the Zen 2.4 Software.

### 4.11. Statistics

Statistical analysis of the data was performed with GraphPad Prism version 9.3.1. The data were subjected to a normality test. Subsequently, the ordinary one-way ANOVA was used, followed by the post hoc Fisher’s LSD test for multiple comparisons, with a family confidence level of 95%. ns: non-significant, *: *p* < 0.05, **: *p* < 0.01, ***: *p* < 0.001, ****: *p* < 0.0001.

## 5. Conclusions

Aneuploidy-inducing gene mutations or oncogenic mutations leading to hyperproliferative signals, as well as excess accumulation of reactive oxygen species (ROS) can induce replication stress (RS) as a tumor protection mechansim. Previous data indicated that telomerase may alleviate the accumulation of RS in response to harmful conditions that induce RS. However, the mechanisms how telomerase suppresses RS is not yet fully understood. In order to address this question, the accurate evaluation of RS in the presence and absence of telomerase is crucial. Therefore, we used telomerase negative normal human fibroblasts (BJ), as well as their telomerase positive counterparts (BJ-hTERT) to compare the suitability of three protein markers (pRPA2, γ-H2AX and 53BP1), which were previously reported to accumulate in response to harmful conditions leading to RS in cells. In summary, we find that pRPA2 is the most consistent and reliable marker for the detection of RS in all tested conditions. Moreover, the results clearly demonstrate the impact of telomerase in alleviating AI-RS, OI-RS, and ROS-induced RS. As RS can promote genome instability, these data support the idea that the downregulation of telomerase in human cells acts as a tumor suppressor mechanism as the cells with aneuploidy-inducing or oncogenic mutations induce senescence or apoptosis. Our data also demonstrate that specific inhibition of ATM and the ATR kinases by small compounds impairs the activation of RS/DDR-induced foci formation in the absence of telomerase but does not interefe with suppression of RS by telomerase. The results may suggest that the induction of RS is prevented by telomerase through the suppression of ATM- and ATR-kinase activation.

## Data Availability

The source data of the presented results, including the supplementary results, are available on request.

## Supplementary Materials

The following supporting information can be downloaded at: https://www.mdpi.com/article/10.3390/cancers14092205/s1, Figure S1: Control experiments showing the knockdown efficiency of the indicated shRNAs, ectopic RASG12V expression and the TRAP Assay indicating telomerase activity, Figure S2: Evaluation of the different markers upon ROS-induced replication stress via Western blot.
