# Supplementary material for: A Comparative Assessment of Replication Stress Markers in the Context of Telomerase
Source: Cancers (Basel). 2022 Apr 28;14(9):2205. doi: 10.3390/cancers14092205 (PMC9103842; doi:10.3390/cancers14092205)

Supplementary Figure S1 A

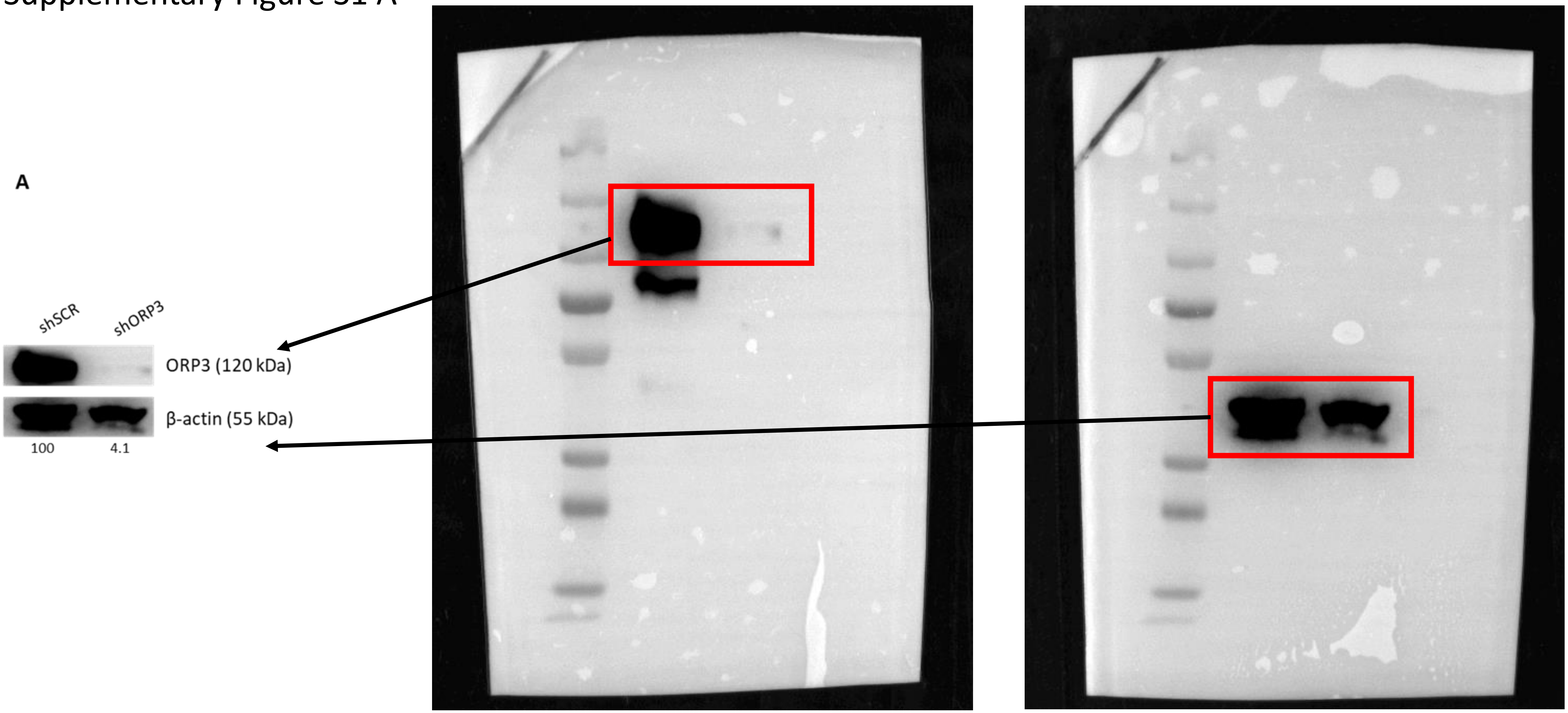

Supplementary Figure S1 B

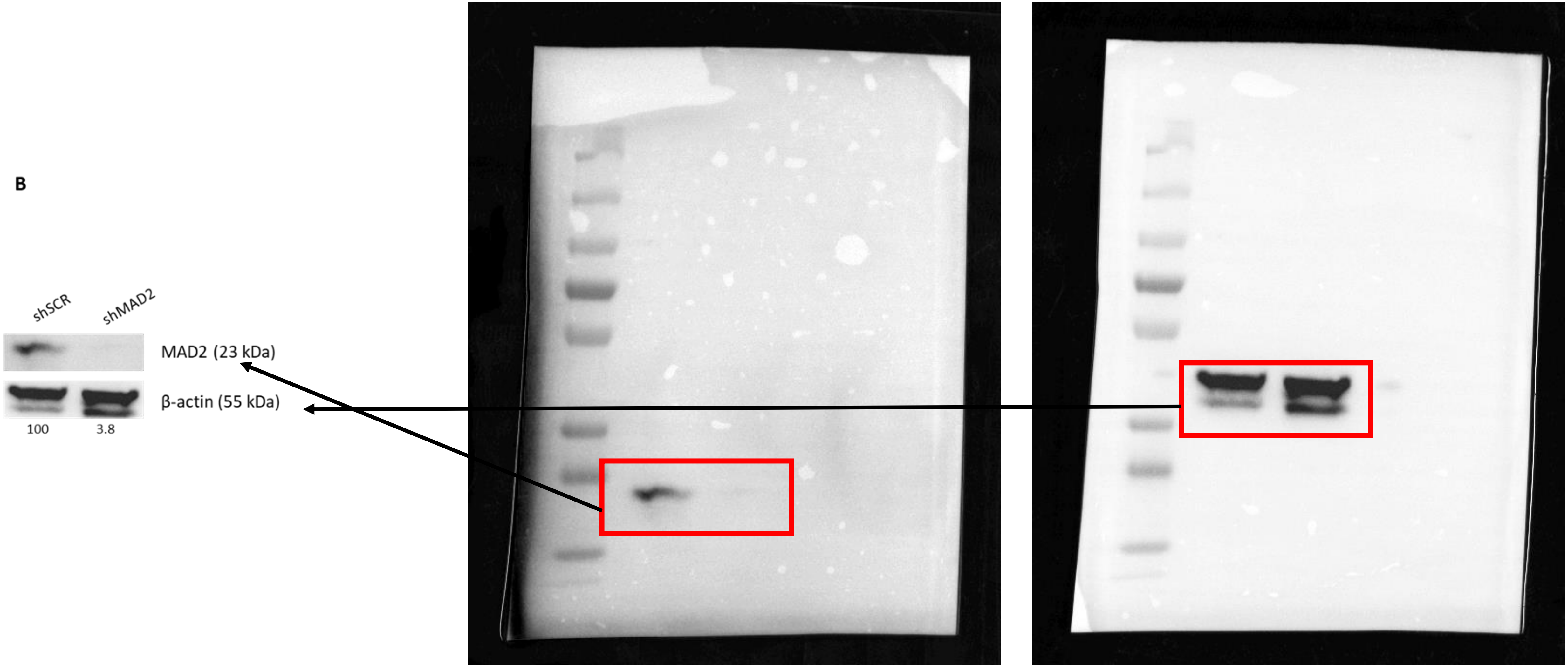

Supplementary Figure S1 C

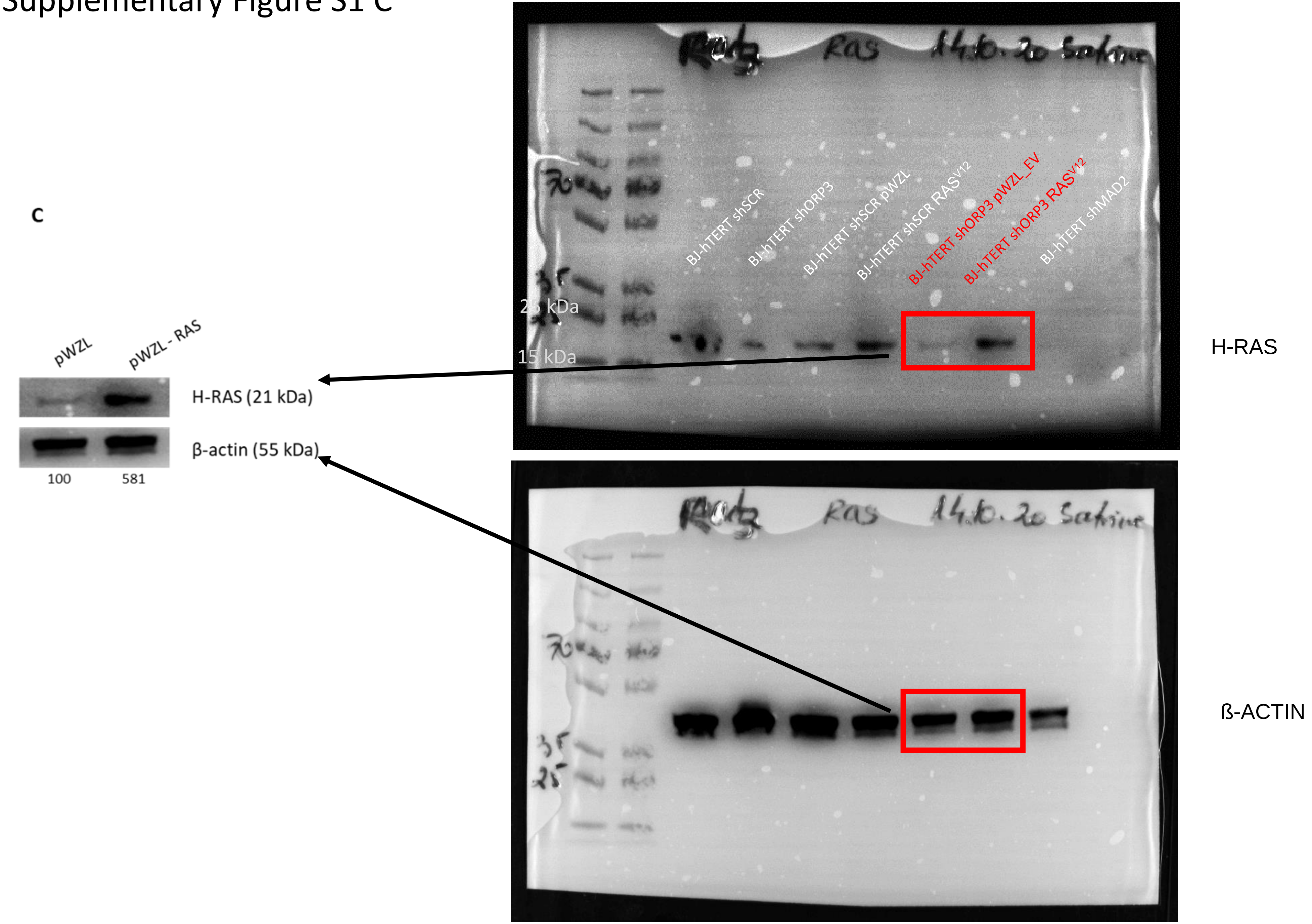

Supplementary Figure S1 D

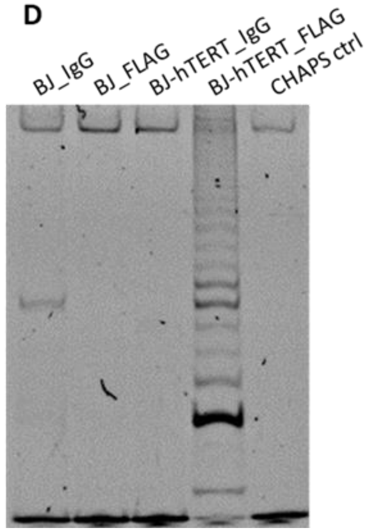

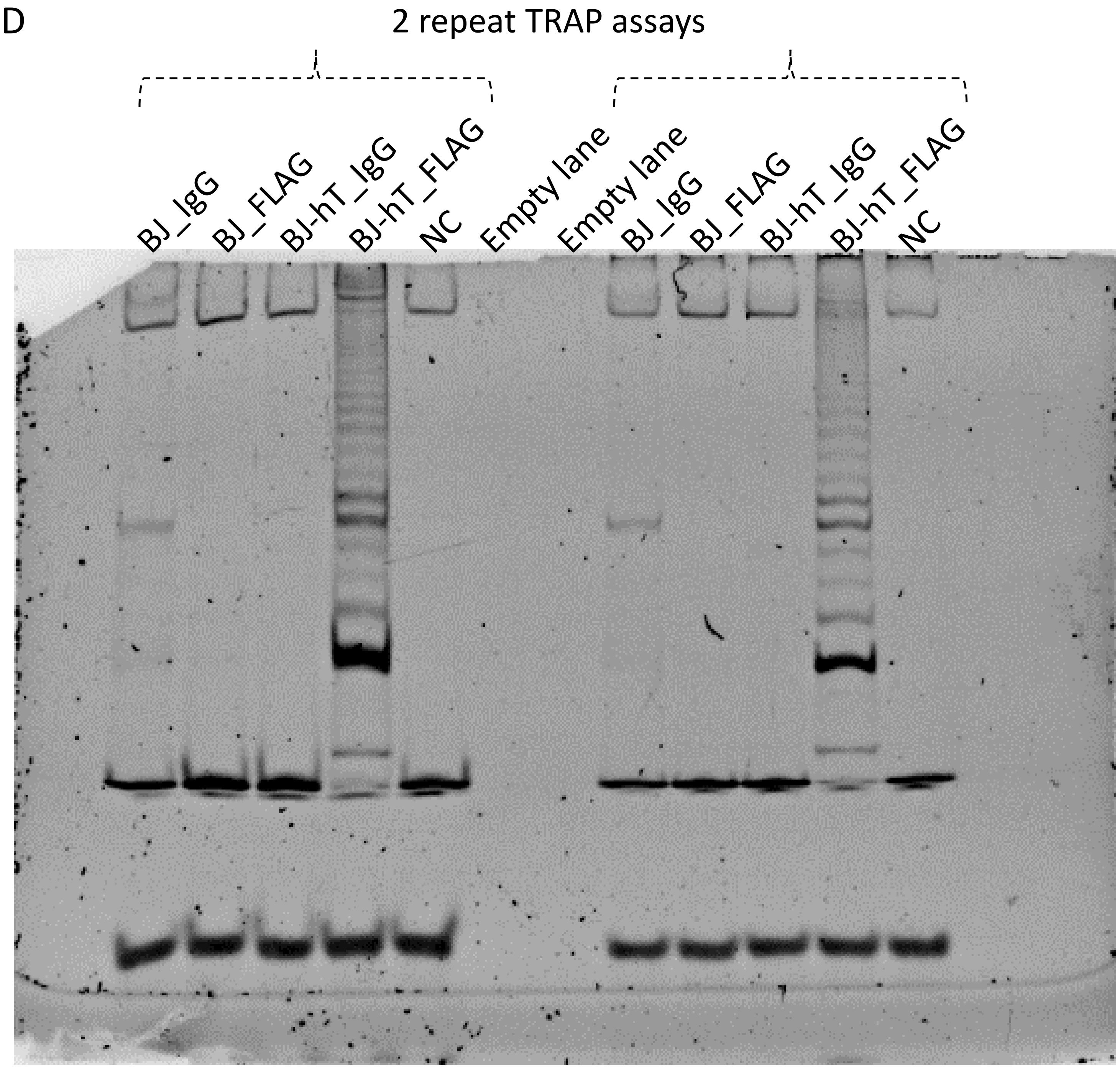

Supplementary Figure S2

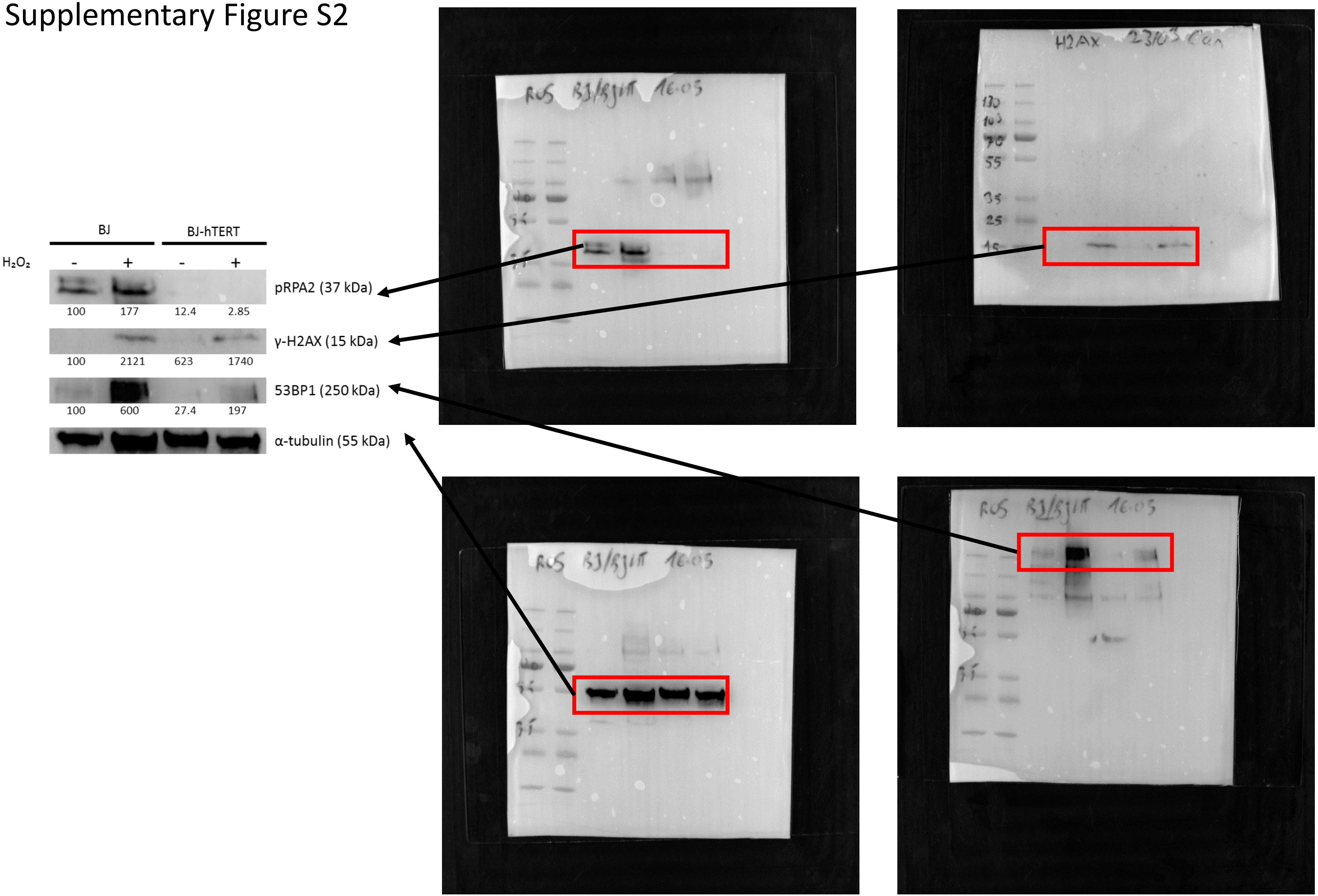

The whole Western blot figure (uncropped blots) of Figure 10

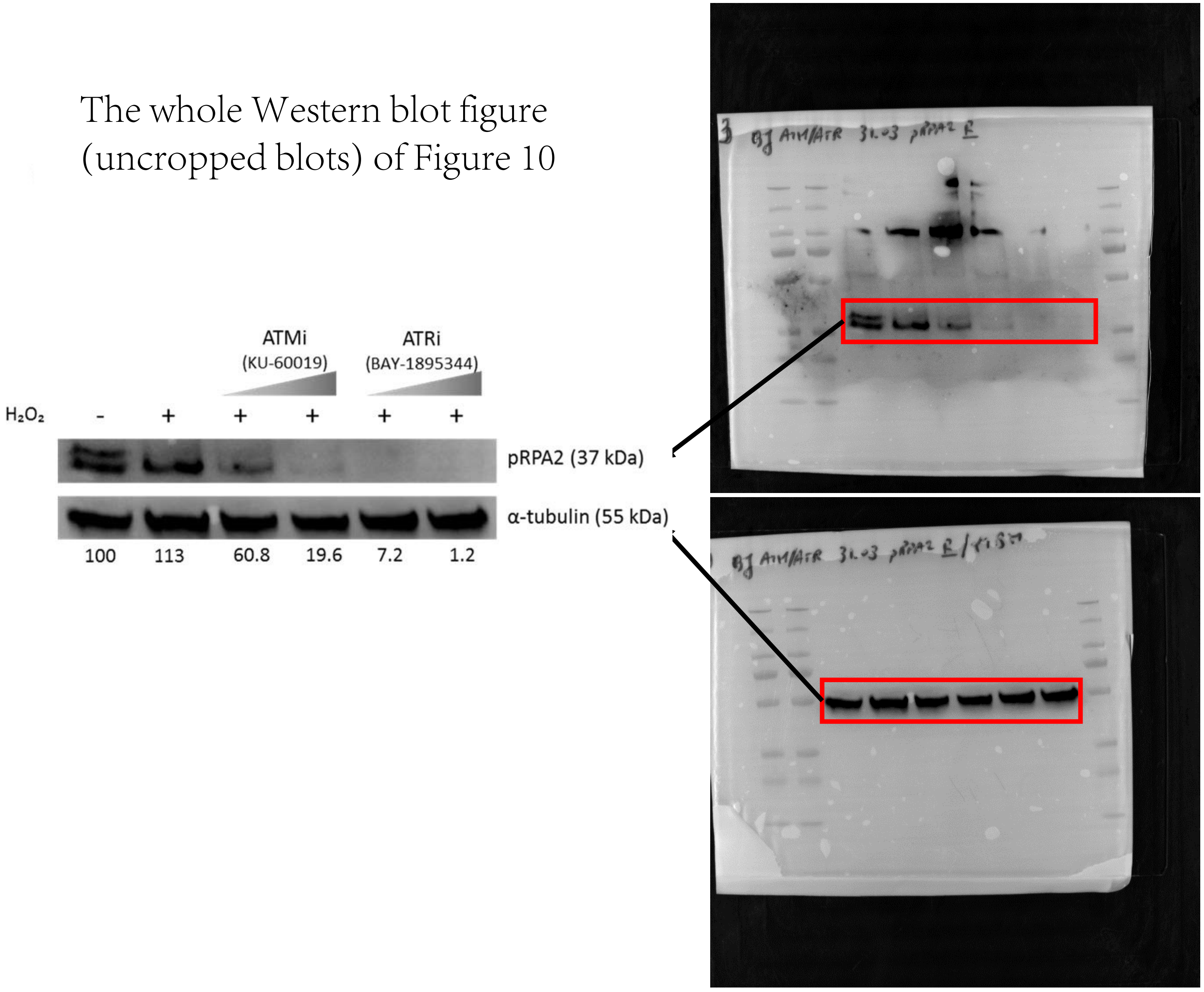

Supplement: Supplementary file 1 [file cancers-14-02205-s001.zip › cancers-1706380-figures.pdf]
