# Supplementary material for: A Comparative Assessment of Replication Stress Markers in the Context of Telomerase
Source: Cancers (Basel). 2022 Apr 28;14(9):2205. doi: 10.3390/cancers14092205 (PMC9103842; doi:10.3390/cancers14092205)
Supplement: Supplementary file 1 [file cancers-14-02205-s001.zip › cancers-1706380-supplementary.pdf]

Supplementary Figure S1

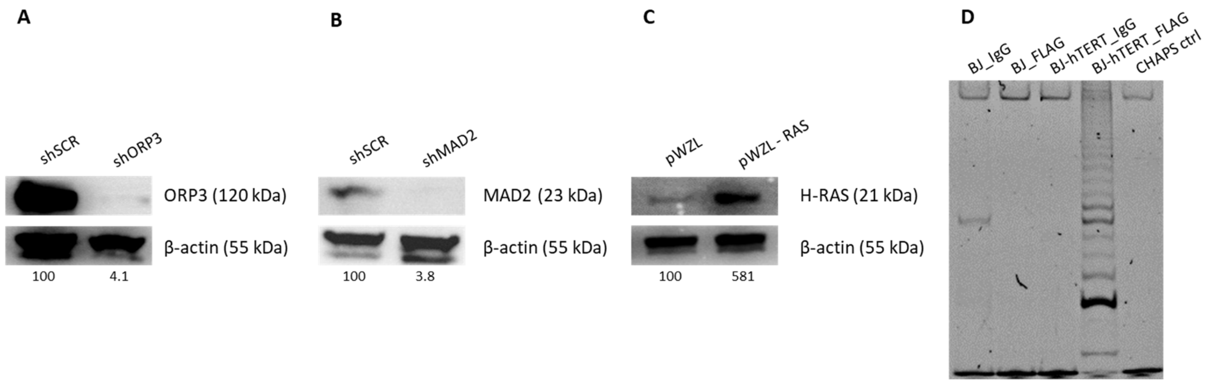

Supplementary Figure S1. Control experiments showing the knockdown efficiency of the indicated shRNAs, ectopic RAS^{G12V} expression and the TRAP Assay indicating telomerase activity. **(A)** A representative western blot showing the knockdown of ORP3 in BJ-hTERT cells. **(B)** A representative Western blot showing the knockdown of MAD2 in BJ-hTERT cells. **(C)** A representative Western blot showing ectopic expression of RAS^{G12V} in BJ-hTERT cells. Intensity ratios of the different bands, normalized to β-actin, and relative to shSCR or pWZL controls are indicated below the blot (in %) **(D)** TRAP assay showing the telomerase activity in BJ-hTERT cells. Nuclear cell lysates from equal number of cells were immune-precipitated with the anti-Flag antibody or IgG control antibody from BJ and BJ-hTERT cells the ladder indicates telomerase activity in BJ-hTERT cells expressing the Flag-tagged TERT protein. CHAPS ctrl indicates the negative control containing CHAPS lysis buffer instead of nuclear extract sample.

Supplementary Figure S2

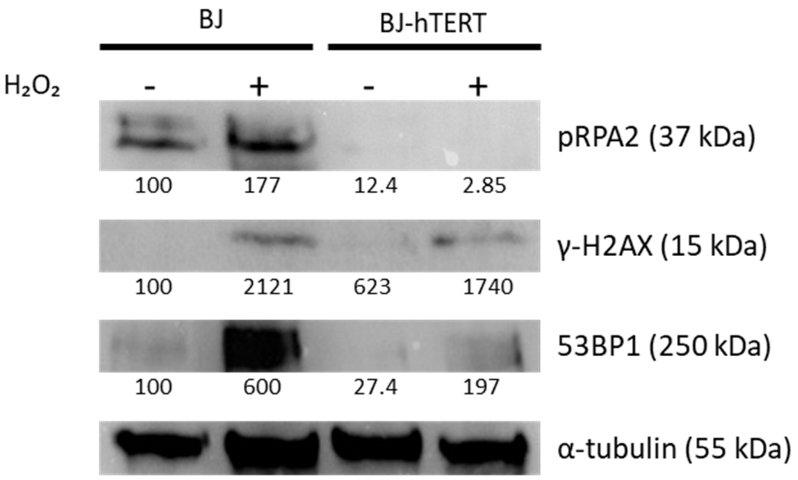

Supplementary Figure S2. **Evaluation of the different markers upon ROS-induced replication stress via Western blot.** In order to determine the total levels of the different markers, we performed Western blot using whole cell lysates from BJ and BJ-hTERT cells after treatment with H_2O_2. Representative Western blots showing the total protein levels of pRPA2, γ-H2AX, and 53BP1 upon H_2O_2-treatment from whole cells lysates of BJ and BJ-hTERT cells. As a loading control, α-tubulin is shown. We observe a clear induction of pRPA2, γ-H2AX, and 53BP1 upon treatment of the BJ cells. Similar to the IF staining, the presence of telomerase reduces the total levels of the different markers. Of note, in accordance to our previous observations, a shift of the pRPA2 upper band is seen in H_2O_2-treated BJ cells. Intensity ratios of the different bands, normalized to α-tubulin, and relative to BJ cells without treatment, are indicated below the blot (in %)
